# Analysis of Dose-response Relationship between BMI and Hypertension in Northeastern China Using Restricted Cubic Spline Functions

**DOI:** 10.1038/s41598-019-54827-2

**Published:** 2019-12-03

**Authors:** Yangming Qu, HuiKun Niu, Lu Li, Meiqi Li, Shoumeng Yan, Meng Li, Shan Jiang, Xiaoyu Ma, Bo Li, Hui Wu

**Affiliations:** 1 Department of Neonatology, The First Hospital of Jilin University, 71 Xinmin Street, Changchun, 130021 China; 2 Department of Epidemiology and Biostatistics, Jilin University School of Public Health, 1163 Xinmin Street, Changchun, 130021 China; 3 Shijiazhuang Pharmaceutical Group Zhongqi Pharmaceutical Technology (Shijiazhuang) Co., Ltd, Shijiazhuang, China

**Keywords:** Hypertension, Risk factors

## Abstract

High body mass index (BMI) was significantly associated with hypertension. The purpose of this study is to investigate the association between BMI and hypertension in people in northeast China. Our study was a cross-sectional study conducted from June to August 2012. According to multistage, stratified cluster sampling, a total of 21435 inhabitants aged between 18 and 79 years in Jilin Province were selected randomly. The prevalence of hypertension was 35.66% overall. After adjusting for potential confounders, the multivariable-adjusted odds ratios for the BMI- hypertension association for overweight and obesity were 2.503 (95% confidence interval = 1.912–2.204) and 4.259 (95% confidence interval = 3.883–4.671). The results of multivariable restricted cubic spline regression analysis showed that there was a non-linear relationship between the continuous change of BMI and hypertension (P < 0.001) after adjusting the confounding factors of different genders and age groups, which indicated that there was an adjusted dose-response association between continuous BMI and hypertension.

## Introduction

Hypertension is a multifactorial disease associated with modifiable and non-modifiable risk factors, among which, modifiable factors are obesity, excessive sodium intake, physical exercise in activity and others, non-modifiable factors are age, sex, ethnicity, and genetics and so on^1^. In 2012, noncommunicable diseases contributed to 68% of worldwide death, and as achronic non-communicable disease, hypertension has become an enormous public health problem due to its high prevalence and low rate of control^1^. The prevalence of hypertension is on the rise across the African continent, and in South Africa, some populations reported rates as high as 46%^2^.

In China, the problem of hypertension challenges the population health because of its high prevalence among adults^3–6^.Approximately 320 million people accounting for nearly 30% of the population 18years old and older have hypertension in China, and among them, <5% have their blood pressure under control^4,6^.

A high-salt diet is clearly associated with hypertension^7^, whereas salt intake is a major source of sodium in the general population^8^. A higher sodium intake can make blood pressure rise, which be defined as sodium sensitivity^9^. Genetic aspect, for example, angiotensin converting enzyme (ACE) gene polymorphisms, as a key element in therenin-angiotensin-aldosterone system, is also associated with hypertension^10^.

In worldwide, the prevalence of overweight and obesity constantly rising and has became a global pandemic. In the United States, the 2011–2012 National Health and Nutrition Examination Survey displayed that approximately 16.9% of youth and 34.9% of adults were diagnosed with obesity^11^. In China, from 1980 to 2013, the combined age-standardized prevalence of overweight and obesity in men and women over the age of 20 was 28.3% and 27.4%, respectively^12^. Several studies have reported that there is a link between blood pressure increase and weight gain^13^. Data from NHANES showed that the prevalence of hypertension in obese people is significantly higher than that in the general population^14^. A prospective analysis also showed that the high prevalence of hypertension in obese patients(>60%) account for 78% of incident of hypertension in men and 64% in women^15^.

Although the classification system of obesity is not exactly identical, body mass index(BMI) is the most commonly used measure, dividing obesity into overweight and various grades^16,17^. Obesity has been shown to be associated with a variety of diseases, such as cancer^18^, hypertension, dyslipidemia, diabetes and cardiovascular disease (CVD)^19–21^. There are few studies to quantify the relationship between BMI levels and hypertension, especially using some intuitive methods such as restricted cubic splines (RCS)^22^. Moreover, nobody has quantified the relationship between BMI levels and hypertension based on the population of Northeast China^15^.

The purpose of this study is to analyse the relationship between BMI and hypertension in people in northeast China. In our study, we used RCS function in dose-response analysis to adjust potential confounding factors to quantify the association between BMI and hypertension. In this way, our study can provide information for people’s health promotion.

## Methods

### Study design

The study which was carried out in Jilin Province was a community-based, cross-sectional study. It starting from June 2012 and ended in August 2012. Research object of the study was the people aged between 18 and 79 years old, and the people must have lived in Jilin Province for more than 6 months. Multistage stratified cluster sampling was used to select research object from cities representative of the distribution of the Jilin population. The detailed sampling procedure had described previously^23,24^. The Ethics Committee of Jilin University School of Public Health approved the study (Reference Number: 2012-R-011). Before participating in this survey, all participants had provided informed consent.

### Definitions

BMI was defined as the ratio of body weight (kilograms) to the square of height (meters). Based on the Chinese criteria of weight for adults, BMI < 18.5, 18.5 ≤ BMI < 24.0, 24.0 ≤ BMI < 28.0,BMI ≥ 28.0 were respectively defined as underweight, normal, overweight and obese^22^. Hypertension was defined as systolic ≥ 140 mmHg and/or diastolic ≥ 90 mmHg following the Chinese Hypertension Prevention Guide (2010 Revised Edition).According to WHO criteria(1999): Through glucoseoxidase method, FPG 7.0 mmol/L and/or 2hPG11.1 mmol/L were regarded as diabetes. Abnormal blood lipids (TC ≥ 5.18 mmol/L or TG ≥ 1.70 mmol/L or HDL-C < 1.04 mmol/L or LDL-C ≥ 3.37 mmol/L) was diagnosed as hyperlipidemia based on the criteria of the “Chinese Guidelines on Prevention and Treatment of Dyslipidemia in Adults”.

Occupations were classified into 3 groups: mental labor, manual labor, and retirees or unemployed. Mental labor was defined as the person who was managers, administrators, or technicians. The people who worked in the manufacturing industry, agriculture, or service industry were classified into manual labor. According to average monthly household, household incomes were classified into6 categories: below 500 yuan, 500 to <1000 yuan, 1000 to <2000 yuan, 2000 to <3000 yuan, 3000 to <5000 yuan, ≥2000 yuan. Smoking was defined as never, ever (smoking at least 100 cigarettes in their lifetime but did not smoke at all), or current. Drinking habits provided two options–“0” and “1”. “0” was defined as no smoking, and “1” was defined as smoking. Exercise habits were classified into 3 categories based on exercise frequency. The exercise frequency of ≥3times a week was defined as “often exercise”, once or twice a week was defined as “sometimes exercise”, the others were defined as “never or rare exercise”.

### Statistical analysis

Demographics are presented as numbers and frequency distributions for categorical variables as appropriate. Univariate logistic regression analyses were used to determined significant differences between hypertension and nohypertension individuals. The association between BMI and hypertension was investigated by using unconditional multivariable logistic regression models and models which adjusted for age, gender, education level, and other variables, and to evaluate the potential confounding effects among risk factor variables. Multivariable-adjusted odds ratios(ORs) and their 95% confidence intervals (CIs) in 3 different logistic regression models were calculated by the SPSS statistical package, version 19.0 (IBM Corp, Armonk, NY) independently. RCS were used to detect the possible nonlinear dependency of the relationship between the risk of hypertension and BMI levels, using 4 knots at prespecified locations according to the percentiles of the distribution of BMI, 18 kg/m^2^, 22 kg/m^2^, 25 kg/m^2^, 27 kg/m^222^. Stata 12.0 (StataCorp, College Station, TX) was used to carry out the above-mentioned dose-response analyses. A significance level of P < 0.05 (2-tailed tests) was used.

## Results

### Characteristics of the study population

A total of 21,435 samples participated in our study, screened for data and eventually included in the study for 20,839 by deleting the missing height/weight values. Among the 20,839 participants, the prevalence of hypertension was35.66% (7,431/20,839), with a number of 3,836 for male and 3,595 for female. The mean age was (47.27 ± 13.34) years. Compared with hypertension participants, the no hypertension individuals had a lower BMI (23.46 ± 3.55 kg/m^2^ vs 25.58 ± 3.65 kg/m^2^, P < 0.001).

The key variables for hypertension and no hypertension considered in our study are listed in Table 1. Significant differences were found in variables gender,age, residential areas, education, marital status, occupation, household income, smoker, drinking, and exercise (P < 0.001).

**Table 1:** Characteristics of the Study Population (N = 20839), 2012,Jilin Province, China.

Variables	Hypertension (7431)	No hypertension (13408)	OR(95%CI)	χ^2^	*P* Value
Gender				83.44	<0.001
Male	3836	6037	1.000		
Female	3595	7371	0.768(0.725–0.812)		
Age(years)				2674.798	<0.001
18–34	417	3498	1.000		
35–44	1101	3648	2.532(2.241–2.860)		
45–54	2255	3372	5.610(5.002–6.291)		
55–64	2351	2132	9.250(8.227–10.401)		
65–79	1307	758	14.464(12.633–16.560)		
Residential areas				32.925	<0.001
Urban	3629	7104	1.000		
Rural	3802	6304	1.181(1.116–1.250)		
Education				407.698	<0.001
Primary school					
or less	2697	3446	1.000		
Junior high school	2142	3835	0.714(0.663–0.768)		
Senior high school	1799	3550	0.647(0.600–0.699)		
University or above	793	2577	0.393(0.358–0.432)		
Marital status				38.898	<0.001
Married	6520	11341	1.000		
Single/divorced/widowed	911	2067	0.767(0.705–0.834)		
Occupation				484.427	<0.001
Mental workers	1119	3103	1.000		
Manual workers	3959	7730	1.420(1.313–1.536)		
Others	2353	2575	2.534(2.320–2.768)		
Household income				204.901	<0.001
<500	1813	2424	1.000		
500~	1489	2392	0.832(0.762–0.910)		
1000~	2391	4449	0.719(0.664–0.777)		
2000~	1154	2677	0.576(0.526–0.632)		
3000~	584	1466	0.533(0.475–0.597)		
Smoker				156.842	<0.001
Never	4248	8463	1.000		
Ever	818	858	1.899(1.714–2.105)		
Current	2365	4087	1.153(1.083–1.2271)		
				41.831	<0.001
No	4891	9407	1.000		
Yes	2540	4001	1.221(1.149–1.297)		
Exercise				422.1796	<0.001
Often	2842	3369	1.000		
Occasionally	1423	3584	0.471(0.4352–0.509)		
Never	3166	6455	0.581(0.545–0.621)		
BMI				1401.501	<0.001
Normal	2433	7058	1.000		
Underweight	114	827	0.400(0.327–0.489)		
Overweight	3125	4177	2.170(2.033–2.317)		
Obesity	1759	1346	3.791(3.483–4.126)		

Abbreviations: OR, odds ration; CI, confidence interval; BMI, body mass index (kg/m^2^); DM, diabetes mellitus.

### Univariate and multivariate logistic regression analyses for investigation of the association between BMI and hypertension

Table 2 showed the results of univariate and multivariate logistic regression analyses. With the BMI classification as the only covariate, the ORs applied by unadjusted univariate logistic regression model were 0.40(95%CI = 0.33–0.49) for underweight, 2.17(95%CI = 2.0–2.32) for overweight, 3.79(95%CI = 3.48–4.13) for obesity (*P* value < 0.001). After adjustment for age and gender, the adjusted odds ratios showed a consistent association between BMI classification and hypertension across the model. In a fully adjusted model, the impact of BMI was slightly diminished when adjusting for residential areas, education, marital status, occupation, household income, smoking and drinking status, exercise. Although the association of BMI classification with hypertension prevalence was reduced with sequential adjustments, BMI showed a significant and clear gradient from the lowest underweight to the highest obesity level.

**Table 2:** Logistic regression analyses of the association between BMI and hypertension, 2012, Jilin Province, China.

Model Adjustment	BMI Classification	SE	Wald	*P*	OR (95% CI)
Univariate Analysis	<18.5~	0.103	79.753	<0.001	0.400(0.327–0.489)
	18.5~		1315.667		Ref (1. 000)
	24.0~	0.033	539.909	<0.001	2.170(2.033–2.317)
	28.0~	0.043	952.685	<0.001	3.791(3.483–4.126)
Adjust Model^a^	<18.5~	0.111	67.019	<0.001	0.403(0.324–0.501)
	18.5~		1194.594		Ref (1. 000)
	24.0~	0.036	396.814	<0.001	2.046(1.907–2.195)
	28.0~	0.047	949.563	<0.001	4.237(3.865–4.644)
Adjust Model^b^	<18.5~	0.112	66.095	<0.001	0.404(0.325–0.502)
	18.5~		1178.959		Ref (1. 000)
	24.0~	0.036	394.961	<0.001	2.503(1.912–2.204)
	28.0~	0.047	943.510	<0.001	4.259(3.883–4.671)

Model^a^: adjusted for baseline gender and age.

Model^b^: adjusted for baseline gender, age, residential areas, education, marital status, occupation, household income, smoking and drinking status, exercise.

### Dose-response relationship between BMI and hypertension

Based on the stratification of gender and age, we used RCS model with 4knots to simulate the relationship between BMI and the risk for hypertension. Significant nonlinear dose-response association was showed in the relationship between BMI and the risk of hypertension (all P value of nonlinear < 0.001). And dose-response relationship analysis showed that with the continuous change of BMI, the association strength of hypertension increased nonlinearly.

**Figure 1:**
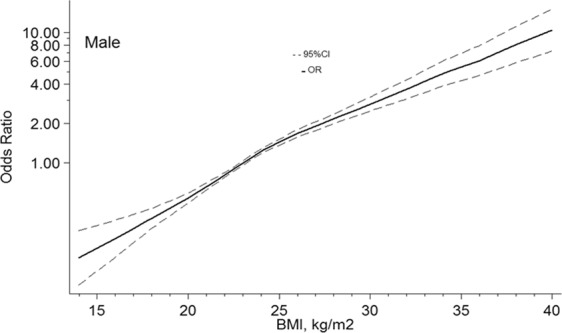
Association between BMI and the risk of hypertension in males, allowing for nonlinear effects, with 95%CI. The model shows ORs compared with BMI 23 kg/m^2^, adjusting age, drinking, and residential areas. BMI, body mass index; CI, confidence interval; OR, odds ratio.

**Figure 2:**
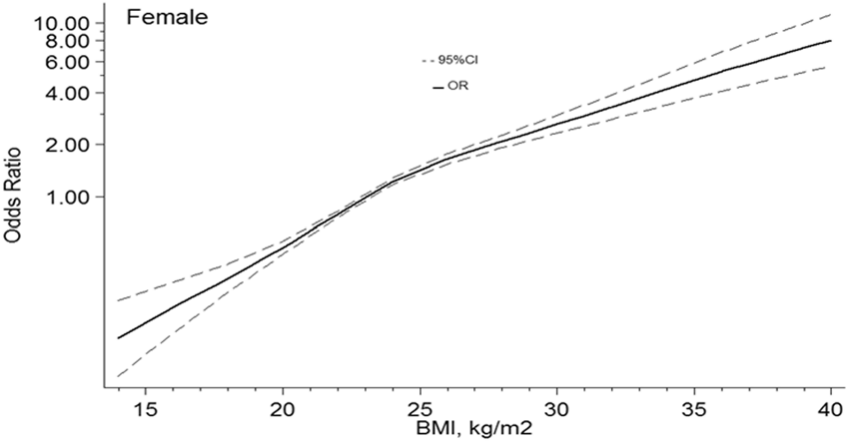
Association between BMI and the risk of hypertension in female, allowing for nonlinear effects, with 95%CI. The model shows ORs compared with BMI 23 kg/m^2^, adjusting age, smoker, and residential areas. BMI, body mass index; CI, confidence interval; OR, odds ratio.

In males, with 23 kg/m^2^ of BMI as a reference, the ORs and 95%CIs of the 4knots of BMI were 0.37(0.31–0.45) for18kg/m^2^, 0.81(0.77–0.84) for 22 kg/m^2^, 1.46(1.36–1.55) for 25 kg/m^2^, 1.91(1.78–2.06) for 27 kg/m^2^. In females, ORs and 95%CIs of the 4 points of BMI at 18 kg/m^2^, 22 kg/m^2^, 25 kg/m^2^, 27 kg/m^2^ were 0.34(0.28–0.41), 0.80(0.76–0.83), 1.46(1.36–1.55), and 1.86(1.73–2.01). Figures 1 and 2 showed the nonlinear dose-response association, with confounders being adjusted.

**Figure 3:**
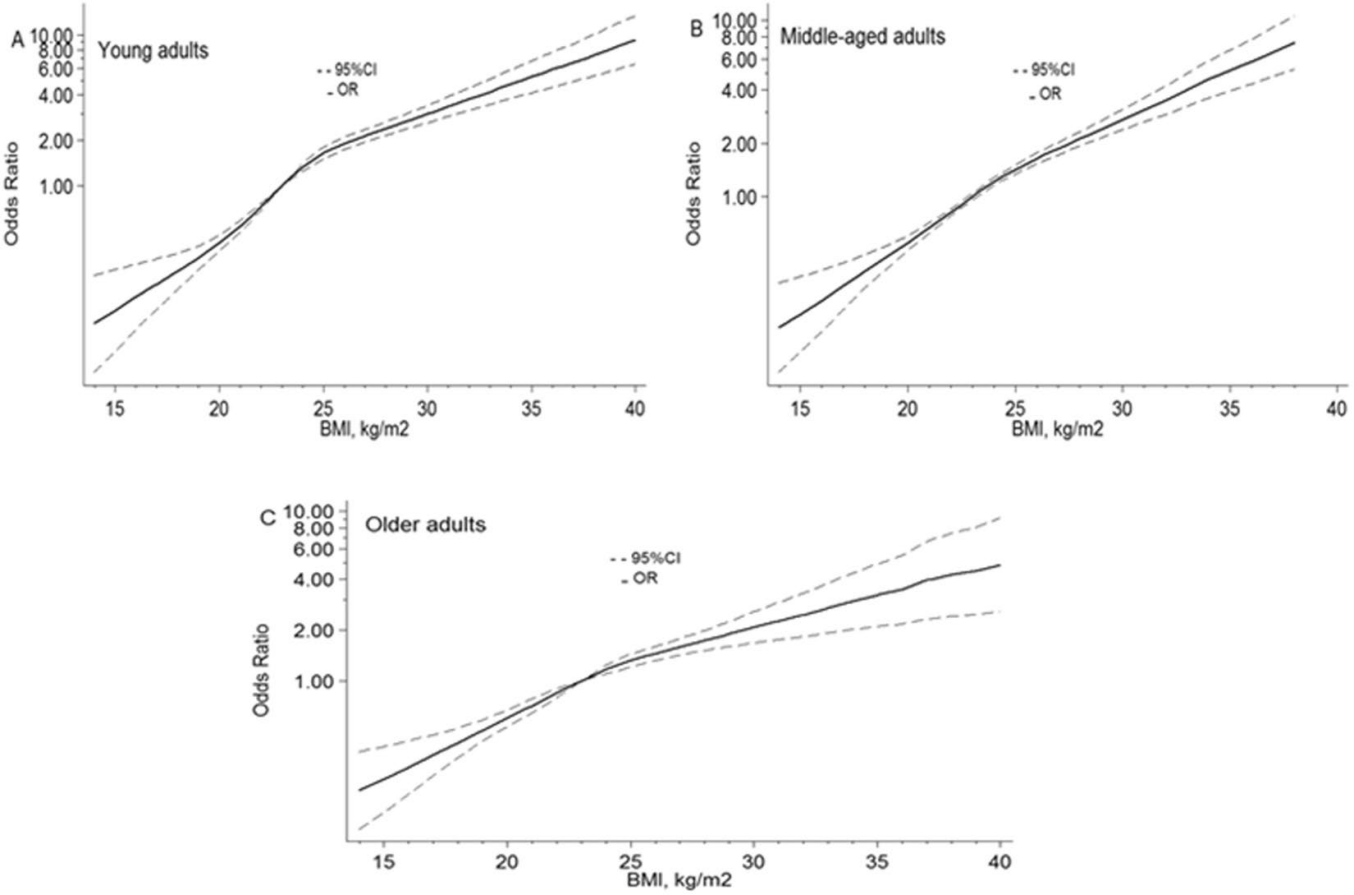
Association between BMI and the risk of hypertension in female, allowing for nonlinear effects, with 95%CI. (**A**) Young adults. Using 4 knots restricted cubic spline for BMI model adjusted gender, residential areas, occupation, smoker, drinking exercise, marital status, and education. (**B**) Middle-aged adults. Using 4 knots restricted cubic spline for BMI model adjusted gender, smoker, drinking, and exercise. (**C**) Older adults. Using 4 knots restricted cubic spline for BMI model adjusted gender, smoker, and exercise.BMI, body mass index; CI, confidence interval; OR, odds ratio.

Similar relationships between hypertension risk and BMI were found in different age groups when using 4 knots (all P value of nonlinear < 0.001; Fig. 3). In young adults (18–44 years old), the points of BMI at 18 kg/m^2^ and 22 kg/m^2^ were, whereas 25 kg/m^2^ and 27 kg/m^2^ were 0.27(0.21–0.36), 0.73(0.69–0.77), 1.65(1.51–1.81), and 2.14(1.95–2.36). For middle-aged adults (45–59 years old) and older adults (65–79 years old), the ORs (95%CIs) for hypertension risk were for BMI at 18 kg/m^2^were 0.38(0.30–0.47) and 0.43(0.35–0.53), at 22 kg/m^2^ were 0.81(0.77–0.85) and 0.85(0.80–0.90), where as at 25 kg/m^2^ were 1.43(1.34–1.53) and 1.32(1.21–1.44), at 27 kg/m^2^ were 1.87(1.73–2.02) and 1.59(1.43–1.78). All age groups are referenced to BMI 23 kg/m^2^.Three groups adjusted forpotential confounders that were tailored to the specific population.

## Discussion

In the population-based study of Jilin Province in Northeastern China, our study reported the prevalence of hypertension was 35.66% including previously diagnosed hypertension and newly diagnosed hypertension. Regardless of gender or age, after adjusting for confounding factors, continuous changes in BMI are significantly associated with the risk of hypertension. And dose-response relationship analysis showed that with the continuous change of BMI, the association strength of hypertension increased nonlinearly.

On a national scale in China, findings based on different backgrounds’ survey in the past 30 years basically showed an upward trend in the prevalence of hypertension^25^. China’s chronic disease monitoring in 2010 reported that the national prevalence of hypertension was 33.5%, which was lower than the rate of 35.66% reported by our study^26^. Our rate was higher than the rate of 17.2% displayed by the monitoring results of Kunshan city in the same year^22^, but not reached the rate of 38.6% of Jiangsu Province^27^.

As early as 1996, study^19^ showed that the increasing of BMIs can make blood pressure increase, which indicated that there was not only a strong relationship between BMI and hypertension, but also was an association between the continuous variables of BMI and blood pressure. Our study and the study^22^ conducted in kunshan city confirmed that there was nonlinear dose-response relationship between BMI and the risk of hypertension.

Although the mechanism of how obesity caused hypertension was not yet clear, epidemiological evidence had highlighted that there was a consistent correlation between obesity and hypertension, and obesity predisposes hypertension^28^. The pathophysiological explanation of obesity predisposing hypertensionis elevated cardiac output, which perhaps attribute to excess intravascular volume and reduced cardiac contractility^29^. There was evidence suggested that in obesity individual, nutritional status alteration, gut microbiota, sunlight exposure and physical activity increase played an important role in the hypertension or not^30^. Further research investigating dose-response association between BMI and hypertension would be a significant step to decrease the social burden of hypertension.

## Conclusion

Our outcomes demonstrated that dose-response relationship exists between BMI and hypertension, and increased BMI is an independent and adjusted dose-dependent risk factor for hypertension among overweight and obesity participants in China.
